# Immunological characterization and comparison of children with COVID-19 from their adult counterparts at single-cell resolution

**DOI:** 10.3389/fimmu.2024.1358725

**Published:** 2024-08-01

**Authors:** Ran Jia, Zifeng Li, Shiwen Hu, Hailing Chang, Mei Zeng, Pengcheng Liu, Lijuan Lu, Menghua Xu, Xiaowen Zhai, Maoxiang Qian, Jin Xu

**Affiliations:** ^1^ Department of Clinical Laboratory, Children’s Hospital of Fudan University, National Children’s Medical Center, Shanghai, China; ^2^ Department of Hematology and Oncology, Children’s Hospital of Fudan University, National Children’s Medical Center, Shanghai, China; ^3^ Department of Infectious Diseases, Children’s Hospital of Fudan University, National Children’s Medical Center, Shanghai, China; ^4^ Institute of Pediatrics and Department of Hematology and Oncology, Children’s Hospital of Fudan University, National Children’s Medical Center, and the Shanghai Key Laboratory of Medical Epigenetics, International Co-laboratory of Medical Epigenetics and Metabolism (Ministry of Science and Technology), Institutes of Biomedical Sciences, Fudan University, Shanghai, China; ^5^ Shanghai Institute of Infectious Disease and Biosecurity, Fudan University, Shanghai, China

**Keywords:** COVID-19, children, single-cell RNA sequencing, innate immunity, NK cell

## Abstract

**Introduction:**

The immunological characteristics that could protect children with coronavirus disease 2019 (COVID-19) from severe or fatal illnesses have not been fully understood yet.

**Methods:**

Here, we performed single-cell RNA sequencing (scRNA-seq) analysis on peripheral blood samples of 15 children (8 with COVID-19) and compared them to 18 adults (13 with COVID-19).

**Results:**

The child-adult integrated single cell data indicated that children with the disease presented a restrained response to type I interferon in most of the major immune cell types, along with suppression of upstream interferon regulatory factor and toll-like receptor expression in monocytes, which was confirmed by *in vitro* interferon stimulation assays. Unlike adult patients, children with COVID-19 showed lower frequencies of activated proinflammatory CD14+ monocytes, possibly explaining the rareness of cytokine storm in them. Notably, natural killer (NK) cells in pediatric patients displayed potent cytotoxicity with a rich expression of cytotoxic molecules and upregulated cytotoxic pathways, whereas the cellular senescence, along with the Notch signaling pathway, was significantly downregulated in NK cells, all suggesting more robust cytotoxicity in NK cells of children than adult patients that was further confirmed by CD107a degranulation assays. Lastly, a modest adaptive immune response was evident with more naïve T cells but less activated and proliferated T cells while less naïve B cells but more activated B cells in children over adult patients.

**Conclusion:**

Conclusively, this preliminary study revealed distinct cell frequency and activation status of major immune cell types, particularly more robust NK cell cytotoxicity in PBMC that might help protect children from severe COVID-19.

## Introduction

Coronavirus disease 2019 (COVID-19), caused by Severe Acute Respiratory Syndrome Coronavirus 2 (SARS-CoV-2), has become a global pandemic since its first outbreak at the end of 2019 (1). The majority of patients with COVID-19 are mild cases, while about 5–10% develop into severe and even life-threatening illnesses (2–4). Notably, children with COVID-19 present with milder symptoms and are at lower risk of hospitalization and life-threatening complications than adults (1, 5–8), which is also the case in patients infected with SARS-CoV or Middle East Respiratory Syndrome Coronavirus (MERS-CoV) (9, 10). On the contrary, elderly COVID-19 patients presented a much higher fatality rate than the younger populations (11). Hence, aging is closely associated with the severity of COVID-19, for reasons that are not yet fully elucidated.

Understanding why children are less prone to develop severe COVID-19 may help optimize strategies to prevent and cure COVID-19. An excessive and disorganized immune response may lead to acute respiratory distress syndrome (ARDS), overwhelming systemic inflammation, and even death (12–14). According to our previous findings, both the pro-inflammatory cytokine response and cytotoxic lymphocyte (CTL) response of children with COVID-19 are milder compared with adults with the disease (7, 15). In consistence, Morhart et al. highlighted that the increased severity of COVID-19 in adults was attributed to the excessive inflammation induced by cytokine production (e.g., IL-6, IL-17A, IL-10) (16). Besides, Kim et al. recently confirmed that children exhibited higher SARS-CoV-2-specific IgG levels and better neutralization activity compared with adults (17). As for immunocytes, Neeland et al. found that children displayed lower proportions of circulating innate immune cells (e.g., monocytes) than their adult counterparts (18). Although these studies have uncovered valuable factors that might protect children from severe COVID-19 illness, the inherent limitations of the methods they employed (e.g., enzyme-linked immunosorbent assay and flow cytometry assay) precluded them from providing multi-perspective immune profiling. As far, a study at single-cell resolution to compare differences in the immune response of children to adults with COVID-19 is still lacking.

In the present study, to unravel the comprehensive immune features of children with COVID-19, we performed single-cell RNA sequencing (scRNA-seq) analysis on peripheral blood mononuclear cells (PBMCs) of children with COVID-19 and compared their whole transcriptomic profiles with those of adult patients from previously published studies (19, 20). As a result, we found distinct patterns of immune responses in children with COVID-19, such as restrained type I interferon (IFN) response, suppressed monocyte activation, and enhanced NK cell cytotoxicity. Moreover, our findings indicated that the enhanced NK cell cytotoxicity in children might be associated with Notch signaling-mediated cellular senescence. Collectively, our study revealed distinct cell frequencies and activation status of major immune cell types, particularly more robust NK cell cytotoxicity in PBMC that might help protect children from severe COVID-19.

## Materials and methods

### Patients and samples

**Figure 1:**
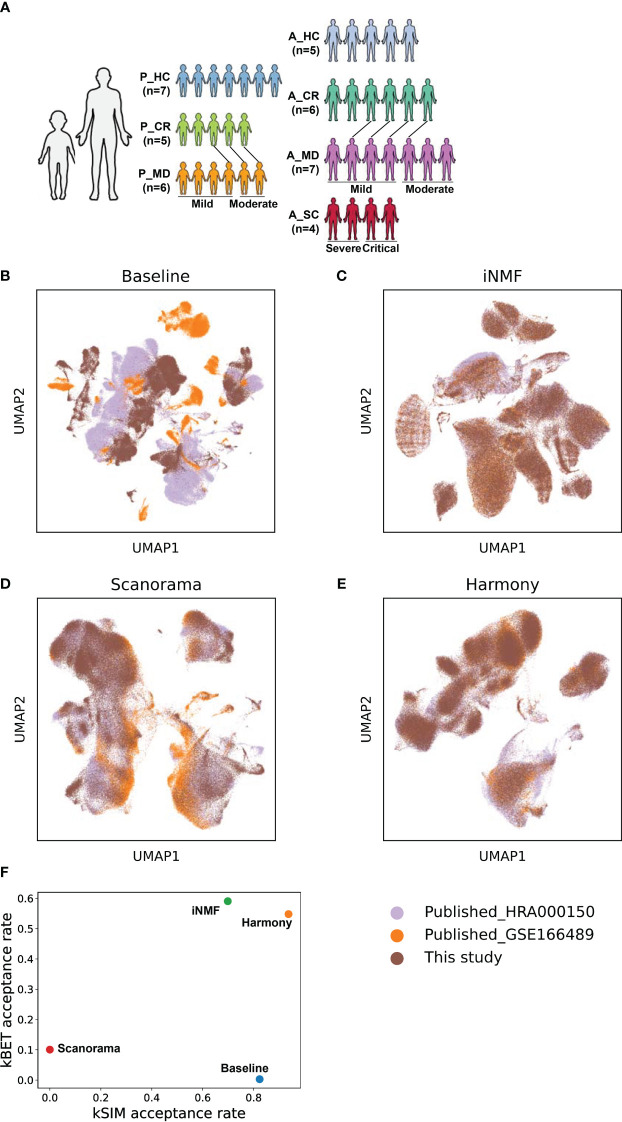
The batch correction effectiveness of different methods. **(A)** Schematic overview of the study design. P, pediatric; A, adult; MD, mild or moderate COVID-19; CR, convalescence; SC, severe or critical COVID-19; HC, healthy controls. The connecting lines between patients indicate those sampled twice. **(B)** UMAP embedding of PBMCs from all samples colored by different datasets before batch correction. **(C)** UMAP embedding of PBMCs from all samples colored by different datasets after batch correction by the *iNMF* method. **(D)** UMAP embedding of PBMCs from all samples colored by different datasets after batch correction by the *Scanorama* method. **(E)** UMAP of classical cell markers expression in some cell subsets after batch correction by the *Harmony* method. **(F)** Scatter plot shows the batch correction effectiveness of different methods assessed by kBET acceptance rate and kSIM acceptance rate.

The scRNA-seq data in this study included two sources: from our own (pediatric patients) and from public databases (adult patients). We collected 13 blood samples from 2 healthy children, 6 with acute COVID-19 (mild-moderate disease), and 5 with convalescent disease. There were 3 children that were sampled at both acute and convalescent phases. Additional 5 healthy children had scRNA-seq data of blood samples that were downloaded from public database Gene Expression Omnibus (accession GSE166489) (20). The scRNA-seq data of 22 blood samples from the 18 adults were requested from public database China Genome Sequence Archive (accession HRA000150) (19), including 5 healthy donors, 11 with acute COVID-19 (7 moderate and 4 severe disease) and 6 with convalescent disease. A total of 4 adults were sampled at both acute and convalescent phases. Overall, a total of 15 children and 18 adults were included in this study (**Table 1**, **Figure 1A**). Demographics and clinical information of these subjects were provided in **Table 1** and **Supplementary Tables 1, 2**. For *in vitro* validation assays, blood samples from an additional 15 COVID-19 patients (5 children and 10 adults) and 6 healthy controls (3 lchildren and 3 adults, presented as HC group) were collected in this study.

**Table 1:** Basic demographics and groups of the study subjects.

		# of Subjects	Sex, male/female	Age, years, median (IQR)
Pediatrics	P_MD *	6	4/2	10 (9~14)
	P_CR	5	2/3	9 (6~13)
	P_HC ^#^	7	4/3	11 (10~13)
Adults	A_MD	7	4/3	37 (35~55)
	A_SC	4	2/2	45 (42~78)
	A_CR	6	4/2	42 (30~49)
	A_HC	5	4/0	39 (31~51)

*P, pediatric; A, adult; MD, mild or moderate COVID-19; CR, convalescence; SC, severe or critical COVID-19; HC, healthy controls.

^#^Data for 5 of 7 P_HC subjects were utilized from published article by Ramaswamy, et al. Immunity. 2021; Volume 54, Issue 5 (https://www.ncbi.nlm.nih.gov/pmc/articles/PMC8043654/bin/mmc1.pdf).

To be noted, the adult patients whose scRNA-seq data were used in the study were diagnosed with COVID-19 in Beijing, China, between 23 January and 15 February 2020 (19), while the children’s samples were collected from COVID-19 patients in Shanghai, China, between 14 March and 7 May 2020 (**Supplementary Table 1**). As evidenced by other studies and the previous work of our team, the complete genome sequence of SARS-CoV-2 prevalent in Shanghai and Beijing during the sampling period was identical or nearly identical to that of Wuhan-Hu-1 (NC_045512.2) (21–23). Moreover, as identified by the World Health Organization (WHO), the first set of variants of concern (VOC) with increased transmissibility and pathogenicity emerged in late 2020 in various global regions, excluding China, such as B.1.1.7 in the UK and B.1.351 in South Africa (24). Therefore, the comparability of the scRNA-seq data between children and adults in our study was validated on the grounds of the similar or even the same pathogenicity of their strains.

All blood samples from patients were collected during the acute phase (within 3 days after the disease onset) or convalescent assessment. These samples were all acquired with written consent from parents of children and adults themselves and approval from the ethics committee of Children’s Hospital of Fudan University [NO. (2020)27]. All procedures performed in studies involving human participants were in accordance with the ethical standards of the institutional and/or national research committee. Following the local control policy of COVID-19 during the sample collection, once patients were identified to be SARS-CoV-2-positive, they needed to be hospitalized and quarantined to limit the transmission of the virus, as was the case with the patients in this study. The clinical information of the patients was presented in **Supplementary Tables 1, 2**. The patients were classified as mild, moderate, severe, or critical disease strictly following the Guidelines for Diagnosis and Treatment of COVID-19 issued by the National Health Commission of China (10th edition) (25). All these patients (our own and from the public) were infected with the virus for the first time, without any co-infection, and all had no COVID-19 vaccination history.

### Preparation of single-cell suspension

PBMCs were isolated from whole blood cells of COVID-19 patients and healthy controls using Ficoll media purchased from Haoyang Bio-Manufacture CO., LTD (Tianjin, China). Briefly, peripheral venous blood samples were centrifuged at 200 g for 10 min to separate sera from blood cells. The blood cells were mixed with RPMI 1640, which were then gently put into a fresh tube added with Ficoll in advance. After a 20 min-centrifugation at 800 g, the layer containing PBMCs was carefully removed to a fresh tube and washed twice with sterile PBS. The PBMCs were resuspended in frozen stock solution with 10% DMSO and 4% FBS and stored at -80°C until use. We utilized the trypan blue solution method for assessing cell viability prior to constructing the single-cell library. The cell viability of all the samples collected in the study exceeded 90%.

### Single-cell 5′ mRNA sequencing and data preprocessing

Suspended PBMCs of COVID-19 patients and healthy controls were loaded into Chromium microfluidic chips and barcoded with a 10X Chromium Controller (10X Genomics). RNA from the barcoded cells was subsequently reverse-transcribed, and sequencing libraries were constructed with reagents from a Chromium Single Cell 5′ Library & Gel Bead Kit (10X Genomics) according to the manufacturer’s instructions. Sequencing was performed with Illumina NovaSeq 6000 sequencer according to the manufacturer’s instructions. The base call files were converted into FASTQs using the Illumina bcl2fastq tool. This study utilized two public datasets, GSE166489 and HRA000150, both were generated by using the Single Cell 5’ Library & Gel Bead Kit from 10x Genomics for library construction. All three datasets underwent sequence alignment to the same human reference genome (GRCh38) through the same processing pipeline. The raw gene expression matrix was preprocessed, and quality was assessed by the python package *Pegasus* (version 1.7.1, https://pypi.org/project/pegasuspy/) unless otherwise stated. First, we included cells with detected genes greater than 500 and less than 6000, and the percent of mitochondrial reads less than 10%. Then we used pegasus.infer_doublets to exclude doublets by predicting potential cell doublets. Finally, a total of 266,914 cells, including 149,695 cells from children and 117,219 cells from adults were retained for downstream analysis. Number of cells corresponding to each category was presented in **Supplementary Table 3**. Cell QC metrics from cell metadata were provided in **Supplementary Table 4**.

### Data integration, batch correction, and cell clustering

The above quality-filtered raw count data from our experiment and public databases were merged into an initial count matrix, followed by logarithmic transformation and normalization, identification of highly variable features, and PCA dimensional reduction, with *pegasus.log_norm* function (norm_count = 1e5) and *pegasus.pca* function in the *Pegasus* package. Data integration and batch correction were then carried out with 3 different methods, namely iNMF (26), Scanorama (27), and Harmony (28) to ensure the best method being pursued that gave great balance in removing technical bias while retaining as much as possible of the biological distance between child and adult patients. This was achieved with two benchmark metrics, namely kBET acceptance rate and kSIM acceptance rate (29, 30). Cell clustering was performed on batch-corrected matrix using Leiden algorithm and visualized using uniform manifold approximation and projection (UMAP) with Pegasus functions *pegasus.umap*.

### Identification of cell types for cell clusters

Cell types were manually assigned to the cell clusters by using putative markers as listed below: *CD3, SELL, CCR7* for naïve T cells, *GZMA, GNLY, PRF1* for activated T cells, *SLC4A10, TRAV1–2* for mucosal associated invariant T (MAIT) cells, *TRGV9, TRDV2* for γδ T cells, *MKI67, PCNA* for proliferative T (pro T) cells, *KLRF1, TYROBP* for natural killer (NK) cells, *MS4A1, CD79A* for B cells, *MZB1* for plasma B cells, *LYZ, CD14* for CD14+ monocytes, *FCGR3A* for CD16+ monocytes, *CD1C* for monocyte-derived dendritic cells, *CLEC4C* for plasmacytoid dendritic cells, and *PPBP, GP9* for platelets.

### Identification of subtypes for major immune cells

Within each immune lineage (Myeloid/NK/B/T), cell subtypes were identified based on functional differences in immune response. To do the subtyping, the count data for monocytes were subset to an independent matrix dataset from the whole count matrix of the PBMCs to further cluster monocytes into subtypes. Normalizing, principal component analysis, and clustering were performed as described above. NK cells, B cells, and T cells were subclustered using the same procedure as for monocytes.

### Gene set score for cell state

The *pegasus.calc_signature_score* function in Pegasus was used to define the gene set score for a predefined gene set of certain biological terms. The function calculated the scores based on the average expression levels of the genes within the predefined gene set in each respective cell. Therefore, a higher gene set score indicates a higher expression level of genes related to that gene set within the cell. Gene set scores were assessed for the following GO terms: response to interferon alpha (GO:0035455), response to interferon beta (GO:0035456), cellular response to type ii interferon (GO:0071346), apoptotic signaling pathway (GO:0097190), leukocyte migration (GO:0050900 GO:0072676), endocytosis (GO:0006897), B cell activation (GO:0042113), T cell activation (GO:0042110), B cell proliferation (GO:0042100), T cell proliferation (GO:0042098), and macrophage proliferation (GO:0061517). These scores will reflect the cell state of a specific biological process with regard to their functional alterations.

### Single-cell and pseudo-bulk differential gene expression and pathway enrichment analysis

To find markers that define cell clusters, differential expression analysis was performed on single-cell matrix data by using the Mann-Whitney *U* test and AUROC-based statistics in *Pegasus*. To generate pseudo-bulk count matrices, the pegasus.pseudobulk function was applied to sum together gene counts of all the cells from the same sample for each cell type. This leads to one gene expression profile for each cell type within each sample and, treating the samples, rather than individual cells, as independent observations for a specific cell type. Also, this enables the testing of differential expression between groups (different disease states) of samples for specific cell types. To find the function of differentially expressed genes (mwu_pval < 0.01, log2(fold change) ≥ +/- 0.5), the R function *enrichKEGG* was applied with the following options: organism = ‘hsa’, keyType = ‘kegg’, pvalueCutoff = 0.05, pAdjustMethod = ‘BH’ in the ClusterProfiler R package (31). To be noted, we believe that it might not be the best way to identify immunologic advantages in children over adults if a P_SC vs. A_SC, or A_MD vs A_SC comparison was carried out, because it was not the severest (A_SC) vs. mildest (P_MD) disease comparison. Hence, to better elucidate the unique benefits of the immune response in children with COVID-19, we ultimately opted to contrast P_MD group with A_SC group in the functional DEGs and KEGG analysis, rather than comparing the patient groups of the same severity.

### *In vitro* IFN-β stimulation assay

PBMCs were isolated from peripheral blood and then adjusted to 10^5^ cells/ml in RPMI 1640 with 10% Fetal Bovine Serum (FBS). Afterward, the cells were stimulated with recombinant human IFN-β (1000 U/ml) or PBS as a control for 6 h at 37°C, 5% CO2 in a 96-well cell culture microplate, and then subjected to quantitative PCR assay as described below.

### CD107a degranulation assay

PBMCs collected from COVID-19 patients and healthy controls were co-cultured with K562 cells at an effector-to-target ratio of 10:1 for 4 h at 37°C and 5% CO2. After the first 1 hour of incubation, the samples were added with GolgiStop (BD #554724). A basal degranulation group (PBMCs without K562 stimulation) was also prepared. During the co-culture, cells were labeled with PE-anti-CD107a antibody (BD #555801). Afterward, the cells were surface-stained with APCCy7-anti-CD20 (Biolegend #302314), V450-anti-CD56 (BD #560360), and FITC-anti-CD3 (BD #555916) to distinguish NK cells from other cells. Then, the samples were tested by flow cytometry (BD FACSCanto II), and raw data were analyzed using FlowJo software (V10.9.0). Results are reported as ΔCD107a^+^ (CD107a^+^ cells % of K562 stimulated samples - CD107a^+^ cells % of unstimulated samples).

### Quantitative real-time PCR

The total RNA of PBMCs was extracted using TRIzol reagent (Life Technologies) following the manufacturer’s protocol, followed by cDNA synthesis using PrimeScript RT-PCR Kit (Takara #RR014A) according to the manufacturer’s instructions. Quantitative PCR (qPCR) was performed using SYBR Premix Ex Taq (Takara #RR420A) on the Applied Biosystems 7500 Real-Time PCR System. Relative mRNA levels were calculated by applying the 2−ΔΔCt method using GAPDH as a reference housekeeping gene. The sequences of primers used were listed in **Supplementary Table 5**.

### Statistics

In the comparison of cell percentage ratios, the partially overlapping samples *t*-test was used for analyzing the P_MD/P_CR group, and the two-sided unpaired Mann–Whitney *U*-test was used for the analysis of other groups. In the comparison of gene set scores, the Wilcoxon test and multiple comparison was employed and the *p* value was adjusted by using the Benjamini–Hochberg procedure. The differentially expressed genes were defined as having an absolute value of log2-fold change of expression greater than or equal to 0.5 with Benjamini-Hochberg adjusted *p* value < 0.01 (two-sided unpaired Mann–Whitney *U*-test).

## Results

### Comparable scRNA-seq data were achieved through effective batch correction and integration between child and adult datasets

The schematic overview of the study design was shown in **Figure 1A**. The scRNA-seq data of pediatric samples were obtained from our own, while those of adult samples were acquired from the public databases (19, 20). The scRNA-seq data sets of all samples were batch-corrected and integrated on quality filtered 266,914 cells as described in the Materials and methods section. Specifically, 149,695 cells from children (P_HC: 61,988 cells, P_CR: 43,709 cells, P_MD: 43,998 cells) and 117,219 cells from adults (A_HC:22,329 cells, A_CR: 36,469 cells, A_MD: 35,708 cells, A_SC: 22,713 cells), were retained for the analysis. As shown in **Figures 1B–E**, the cell distributions varied on UMAPs by different batch removal methods. Determined by the benchmark metrics kBET and kSIM as shown in **Figure 1F**, Harmony method performed well over the other two methods in this study. It yielded the optimal balance in terms of effective batch mixing and preservation of biological relationships (**Figure 1F**), and comprehensive coverage across samples of different disease groups (**Supplementary Figure 1**). Basically, kBET measures whether batches are well-mixed in the local neighborhood of each cell, whereas kSIM measures whether cells of the same pre-annotated cell type are still close to each other in the local neighborhoods after batch correction. Then acceptance rate is the percent of cells with kBET (or kSIM) *p*-values ≥ 0.05 (29, 30). The higher, the better (29, 30).

In addition, unsupervised hierarchical clustering analysis was performed with pseudo-bulk count matrices for samples within the adult and pediatric groups separately (**Supplementary Figure 2**). The clustering analysis indicated the similarity in transcriptomic profiles between mild and moderate patients in both child and adult data sets. Thus, the mild and moderate cases for children and adults were combined accordingly (P_MD and A_MD groups). Considering the small sample size for both severe and critical cases (each = 2), these severe and critical cases were merged as a single group “A_SC” in downstream analysis.

### Distinct frequency advantage of major immune cells in PBMC was seen in child over adult COVID-19

**Figure 2:**
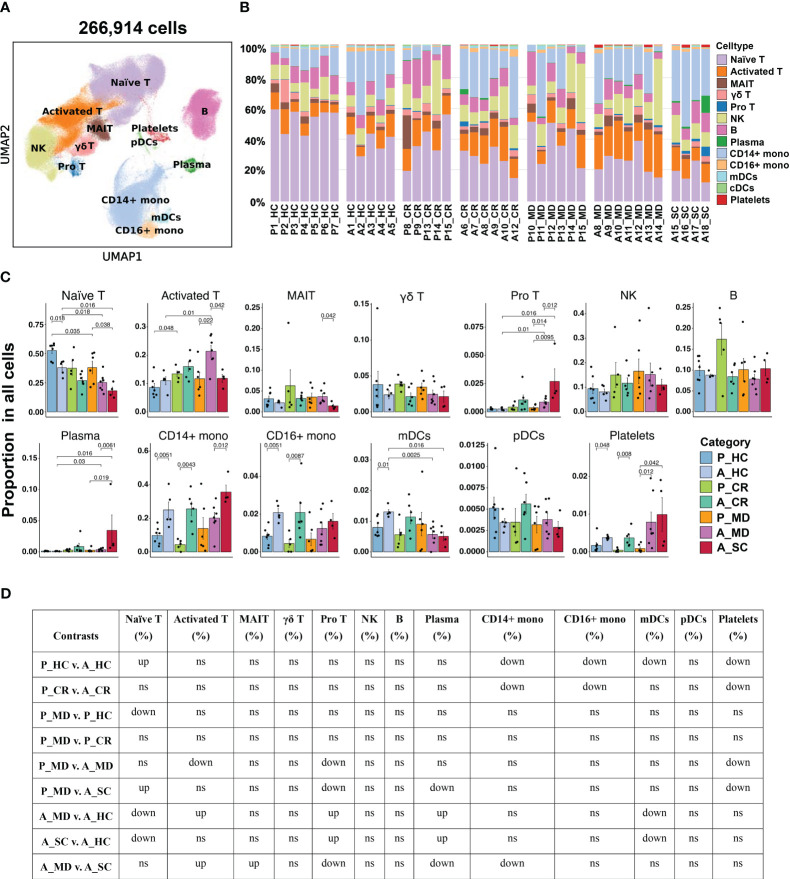
Single-cell transcriptional profiling of PBMCs from healthy donors and patients with COVID-19 in different age groups. **(A)** UMAP embedding of PBMCs from all samples (n=266,914 cells) colored by different cell types. **(B)** Bar plots showing the proportion of each cell type in the total cells in different samples. P, pediatric; A, adult; MD, mild or moderate COVID-19; CR, convalescence; SC, severe or critical COVID-19; HC, healthy controls. **(C)** Bar plots showing the average proportion of each cell type in the total cells colored by donors of different conditions. Error bars represent ± s.e.m. Meaningful differences with *P* < 0.05 are indicated; the partially overlapping samples *t*-test was used for the analysis of P_MD and P_CR group; the two-sided unpaired Mann–Whitney *U*-test was used for the analysis of other groups. **(D)** Significance checkup table for the percentage of major cell types of PBMCs from all samples. ns, non-significant. ns, non-significant. up, significantly higher or up-regulated. down, significantly lower or downregulated.

Overall, 13 major cell types were identified based on putative immune cell marker genes, including naïve T cells, activated T cells, MAIT cells, γδ T cells, pro T cells, NK cells, B cells, plasma cells, CD14+ mono, CD16+ mono, mDCs, pDCs and platelets (**Figures 2A, B**, **Supplementary Figure 3**) (19, 32). Lists of significantly differentially expressed genes (DEGs) in different cell types between children and adults with COVID-19 can be found in **Supplementary Tables 6–9**. Consistent with another report (8), there was a significantly higher percentage of naïve T cells in the samples from healthy children (P_HC) than from healthy adults (A_HC) (*p* = 0.018; **Figures 2C, D**). More importantly, the proportion of naïve T cells showed a downward trend from P_MD group to A_MD group and A_SC group, with P_MD group having a significantly higher proportion of naïve T cells than A_SC group (**Figures 2C, D**). This possibly indicates a decline in naïve T cells with both disease severity and age. Additionally, naïve T cells in A_MD and A_SC groups were lower than that of A_HC group, which is also the case for P_MD and P_HC groups (**Figures 2C, D**). In contrast, pro T cells were significantly lower in P_MD group than in A_MD and A_SC groups (**Figures 2C, D**), indicating a lower vitality of T cells in children. Notably, the percentage of pro T cells in A_SC group is higher than that of A_MD group, possibly suggesting an unfavorable effect on the disease (**Figures 2C, D**). Although the proportion of total B cells in pediatric patients was comparable with that of adult patients, the proportion of plasma cells in P_MD group was significantly lower than that of A_SC group (**Figures 2C, D**). Both A_SC and A_MD groups exhibited a significantly higher proportion of plasma cells than A_HC group (**Figures 2C, D**), while this contrast was not evident in P_MD and P_HC groups, suggesting a more robust humoral response in adult individuals with COVID-19. It is worth noting that the A_SC group had a higher proportion of CD14+ monocytes than A_MD group (**Figures 2C, D**), potentially associated with the exacerbated inflammatory damage in severe/critical COVID-19 cases. Also, the percentages of CD14+ monocytes and CD16+ monocytes in children were lower compared to adults in both healthy and convalescent states (**Figures 2C, D**), possibly indicating that children naturally have fewer functional monocytes. To be added, we noticed a possible up-trend in the proportion of NK cells overall in children over adult counterparts, although there were no significant changes (**Figures 2C, D**). These data indicate that children demonstrate distinct characteristics in immune cell types.

**Figure 3:**
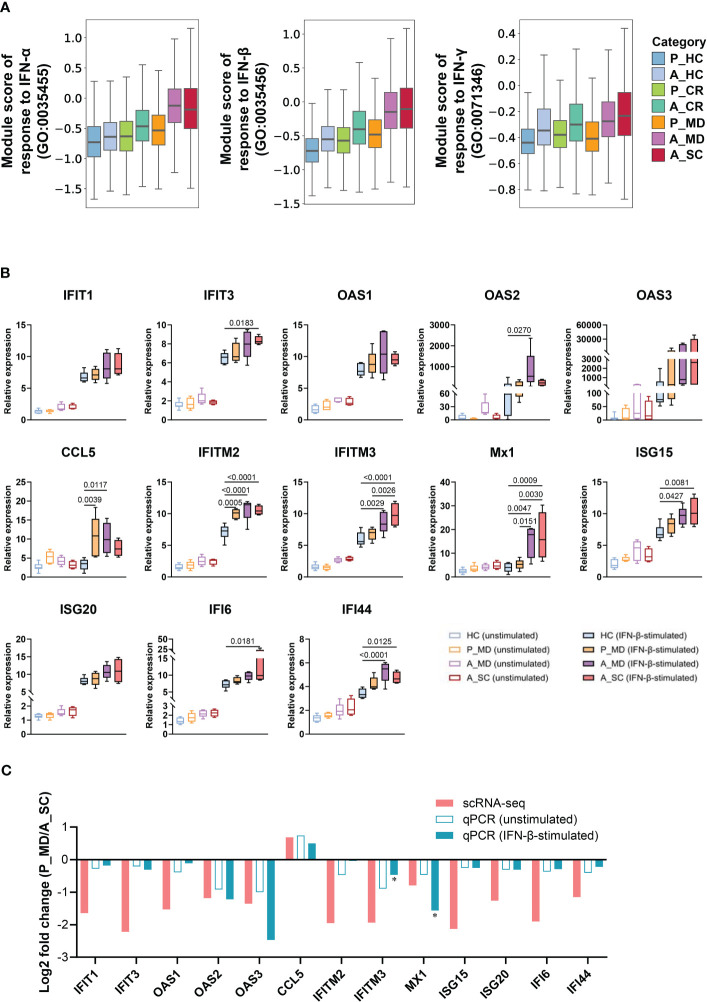
Restrained IFN response in children with COVID-19. **(A)** Box plots of the expression levels of response to IFN in total cells colored by donors of different conditions. P, pediatric; A, adult; MD, mild or moderate COVID-19; CR, convalescence; SC, severe or critical COVID-19; HC, healthy controls. **(B)** PBMCs with or without IFN-β stimulation were subjected to qPCR to detect the expression of ISGs. GAPDH was used as a housekeeping gene. Statistical analysis was conducted using one-way ANOVA. Meaningful differences with *P *< 0.05 are indicated. Error bars represent min to max. IFIT, Interferon induced protein with tetratricopeptide repeats; OAS, 2’-5’-Oligoadenylate synthetase; IFITM, interferon induced transmembrane protein; CCL5, C-C Motif Chemokine Ligand 5; Mx1, Myxovirus resistance 1; ISG, Interferon-stimulated gene product; IFI, Interferon induced protein. **(C)** Validation of scRNA-seq data using qPCR. The fold changes represent the expression changes of each ISG in P_MD group to the A_SC group (determined through scRNA-seq and qPCR). * fold-changes by scRNA-seq were all adj.*p*<0.05 and those with * sign indicate a significance level at *p*<0.05 by qPCR.

Since interferons (IFN) are key anti-viral cytokines in innate immune response and act as an important bridge linking innate and adaptive immune responses (33), we next evaluated the cell state of IFN-associated pathways in different sample groups (**Figure 3A**) and major cell clusters (**Supplementary Figure 4**). Notably, the responses to IFN-α and IFN-β of A_MD and A_SC groups were significantly enhanced in overall cells (**Figure 3A**) and in specific subtypes such as CD14+ monocytes, CD16+ monocytes, and mDCs(**Supplementary Figure 4**), while the responses were much weaker in P_MD group. To further validate this finding, we conducted *in vitro* IFN stimulation assays. As shown in **Figure 3B**, the expression levels of ISGs were mostly upregulated after IFN-β stimulation. With IFN-β stimulation, there were significant elevations in relative expression level for 7/13 (54%) tested genes (i.e., *IFIT3, IFITM2, IFITM3, Mx1, ISG15, IFI6, IFI44*) in A_SC group and 7/13 (54%) tested genes (i.e., *OAS2, CCL5, IFITM2, IFITM3, Mx1, ISG15, IFI44*) in A_MD group, while only 2 genes (i.e., *IFITM2* and *CCL5*) had increased their expression levels significantly in pediatric patients (P_MD) when compared to the baseline (healthy controls). The fold-changes for the gene *IFITM2* in P_MD group were not even greater than those in adult patients. None of the 3 sick groups of samples (P_MD, A_MD, A_SC) had significant gene expression changes from the baseline in unstimulated PBMCs. These findings were in line with the scRNA-seq findings for a restrained IFN response in pediatric patients. Moreover, we made comparisons of fold-change values (ratio of the gene expression levels in P_MD group to A_SC group) for the 13 genes in **Figure 3C**. Two genes, *IFITM3* and *MX1*, which exhibited significant differences in expression levels as determined by qPCR between the P_MD and A_SC groups, were labeled with asterisks (**Figure 3C**). Consequently, a significant correlation was found between scRNA-seq and qPCR methods (r = 0.7565, *p* = 0.0017) (**Figure 3C**), suggesting an overall concordance in gene expression changes across different platforms. These validation findings were in support of a restrained IFN response found in scRNA-seq data from children with COVID-19.

### Less activated monocytes but stronger cytotoxic NK cells in innate immunity were evident in child over adult COVID-19

**Figure 4:**
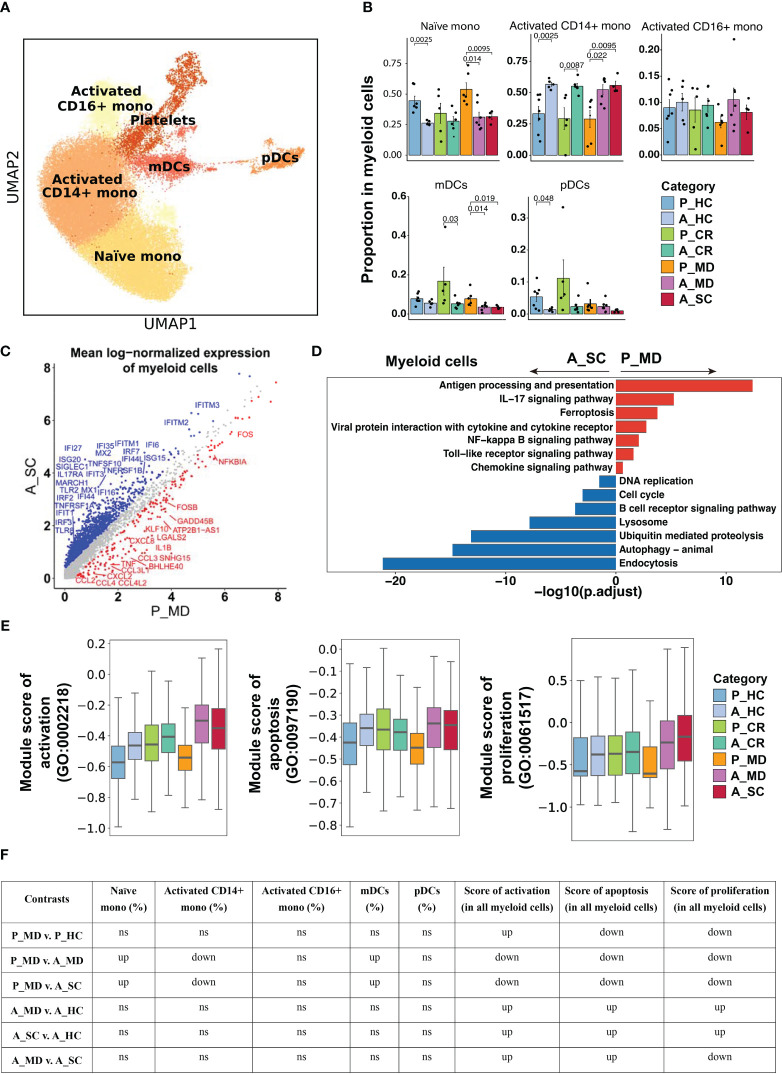
Characteristics of myeloid cell phenotype in different conditions. **(A)** UMAP embedding of myeloid cell phenotype colored by different subsets. **(B)** Bar plots showing the proportion of each cell type colored by donors of different conditions. Error bars represent ± s.e.m. Meaningful differences with *P* < 0.05 are indicated; the partially overlapping samples *t*-test was used for the analysis of P_MD and P_CR group; the two-sided unpaired Mann–Whitney *U*-test was used for the analysis of other groups. P, pediatric; A, adult; MD, mild or moderate COVID-19; CR, convalescence; SC, severe or critical COVID-19; HC, healthy controls. **(C)** Scatter dot plot indicating differentially expressed genes (DEGs) in myeloid cells between P_MD and A_SC group. The cutoffs for DEGs were set to abs[log2(fold change)] ≥ 0.5 with Benjamini-Hochberg adjusted *P* value < 0.01 (two-sided unpaired Mann–Whitney *U*-test). **(D)** Bar plot of Kyoto Encyclopedia of Genes and Genomes (KEGG) pathway analysis using downregulated and upregulated DEGs upon myeloid cells. **(E)** Box plots of the expression levels of GO biological process terms in myeloid cells colored by donors of different conditions. **(F)** Significance checkup table for the percentage of each cell type and score of GO biological process terms in myeloid cells. ns, non-significant. up, significantly higher or up-regulated. down, significantly lower or down-regulated.

**Figure 5:**
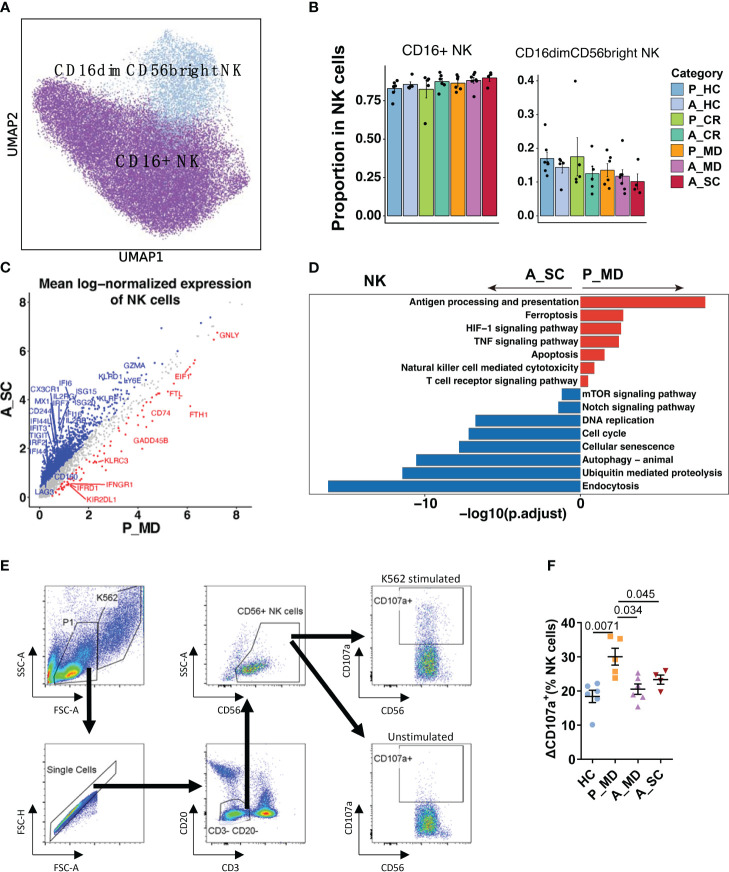
Characteristics of NK cell phenotype in different conditions. **(A)** UMAP embedding of NK cell phenotype colored by different subsets. **(B)** Bar plots showing the proportion of each cell type colored by donors of different conditions. Error bars represent ± s.e.m. Meaningful differences with *P* < 0.05 are indicated; the partially overlapping samples t-test was used for the analysis of P_MD and P_CR group; the two-sided unpaired Mann–Whitney *U*-test was used for the analysis of other groups. P, pediatric; A, adult; MD, mild or moderate COVID-19; CR, convalescence; SC, severe or critical COVID-19; HC, healthy controls. **(C)** Scatter dot plot indicating differentially expressed genes (DEGs) in NK cells between P_MD and A_SC group. The cutoffs for DEGs were set to abs[log2(fold change)] ≥ 0.5 with Benjamini-Hochberg adjusted *P* value < 0.01 (two-sided unpaired Mann–Whitney *U*-test). **(D)** Bar plot of Kyoto Encyclopedia of Genes and Genomes (KEGG) pathway analysis using downregulated and upregulated DEGs upon NK cells. **(E)** Gating strategy of NK cells and CD107a^+^ cells using flow cytometry. **(F)** Comparison of ΔCD107a^+^ NK cells in patients of different groups. Statistical analysis was conducted using one-way ANOVA. Meaningful differences with *P* < 0.05 are indicated. Error bars represent ± s.e.m. ΔCD107a^+^: CD107a^+^ cells % of K562 stimulated samples - CD107a^+^ cells % of unstimulated samples.

Next, we investigated the changes in innate immune responses between pediatric and adult patients with COVID-19. Functional subtypes in the monocyte population (e.g., naïve mono, activated CD14+ mono, activated CD16+ mono, **Figure 4A**, **Supplementary Figure 5**) and in NK cell population (e.g., CD16+ NK, CD16dimCD56bright NK, **Figure 5A**, **Supplementary Figure 6**) were identified using UMAP. In line with the findings in **Figures 2C, D**, children had more naïve mono but less activated CD14+ mono than adults did in both healthy and sick states (**Figures 4B, F**). Enhanced chemokine signaling in children was revealed by DEGs analysis (e.g., *CCL2, CCL3, CCL4, CXCL2, CXCL8*) (**Figure 4C**) and the KEGG pathway enrichment analysis (**Figure 4D**). In line with the suppressed activation, the pathways involving cell vitality (e.g., DNA replication, cell cycle, endocytosis) and the biological processes (e.g., activation, apoptosis and proliferation) in myeloid cells (**Figures 4E, F**), especially in activated monocytes (**Supplementary Figure 7**), were also significantly suppressed in pediatric patients during acute phase. These data are indicative of an inherent lower activation and mild response of monocytes in children with COVID-19.

Considering that children showed suppressed IFN-I response as described earlier in **Figure 3A**, we examined the expression of IFN-I upstream genes in monocytes and pDCs, which are the most rapid and abundant IFN-I producers in respiratory viral infection (34). It was found that both monocytes and pDCs of children with COVID-19 (P_MD) showed a generally lower expression of TLRs and IRFs than that of adult patients (A_MD and A_SC), with monocytes being more prominent (**Supplementary Figure 8**). Considering the strong IFN-I response and highly expressed TLRs and IRFs in the adult severe/critical (A_SC) group, it might be assumable that adult patients had exhausted IFN-I signaling. Collectively, we observed a conserved upstream IFN-I signaling in children with COVID-19.

Similarly, the NK cell subtype analysis defined two subtypes of NK cells (i.e., CD16+ NK and CD16dimCD56bright NK) (**Figures 5A, B**). Despite the insignificant differences, there was a possible up-trend in the proportion of CD16dimCD56bright NK cells in children over adult patients. More notably, several cytotoxicity-related genes (e.g., *FGFBP2, GNLY, IFNG, TNF*) were remarkably upregulated, while some exhaustion-related genes (e.g., *LAG3, CD244, CD160, HAVCR2, TIGIT*) were notably downregulated in NK cells of pediatric patients (**Figure 5C**, **Supplementary Figure 9**). Moreover, an enhanced NK cell cytotoxicity was evident with significantly upregulated pathways, namely NK cell-mediated cytotoxicity pathway and TNF signaling pathways (**Figure 5D**) in children over adult patients. We also found that the cellular senescence pathway was downregulated significantly in children with COVID-19, along with the downregulated Notch signaling pathway (**Figure 5D**). Furthermore, many senescence-associated genes, such as *KIR2DL3, KIR3DL2, USP18, B3GAT1, and KLRC1/D1/K1,* and the genes that were involved in Notch signaling, such as Notch receptor (*Notch1/2*), regulator (*DTX2/3/4*), target (*HEY2, HES7*) and cell cycle (*CCND1, CDKN1B, CDK3/6*) were all markedly downregulated in children with COVID-19 (**Supplementary Figure 10**). It has been reported that impaired cytotoxicity is a hallmark of NK cell senescence (35), and the Notch signaling pathway was reported to contribute to cellular senescence in various cells (36). Hence, it was assumable that the greater cytotoxicity observed in NK cells of children with COVID-19 might be related, at least in part, to a less active Notch signaling-mediated cellular senescence in children. The CD107a degranulation assays further confirmed greater cytotoxicity of NK cells in children (P_MD group) over adult patients with COVID-19 (A_MD and A_SC groups) (**Figures 5E, F**).

### Adaptive immune B and T cell responses were not robust in children as in adult COVID-19

**Figure 6:**
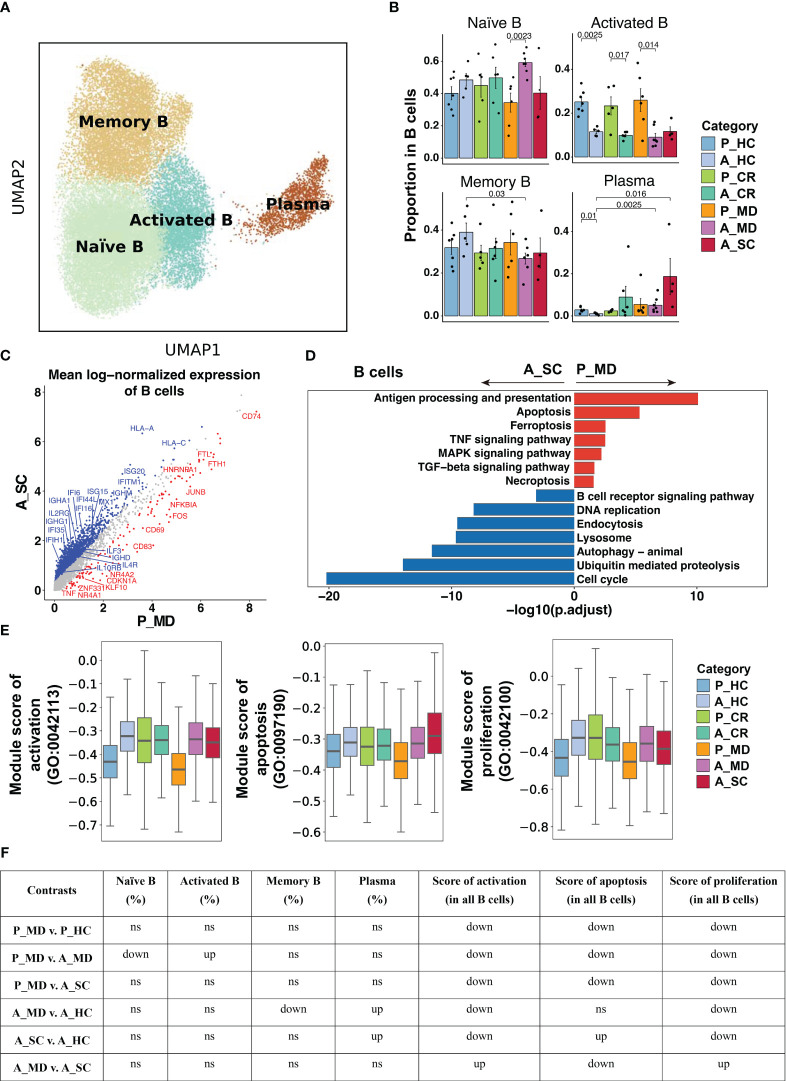
Characteristics of B cell phenotype in different conditions. **(A)**UMAP embedding of B cell colored by different subsets. **(B)** Bar plots showing the proportion of B cell subsets colored by donors of different conditions. Error bars represent ± s.e.m. Meaningful differences with *P* < 0.05 are indicated; the partially overlapping samples *t*-test was used for the analysis of P_MD and P_CR group; the two-sided unpaired Mann–Whitney *U*-test was used for the analysis of other groups. P, pediatric; A, adult; MD, mild or moderate COVID-19; CR, convalescence; SC, severe or critical COVID-19; HC, healthy controls. **(C)** Scatter dot plot indicating DEGs in B cells between P_MD and A_SC group. The cutoffs for DEGs were set to log2(fold change) +- ≥ 0.5 with Benjamini-Hochberg adjusted *P* value < 0.01 (two-sided unpaired Mann–Whitney *U-*test). **(D)** Bar plot of Kyoto Encyclopedia of Genes and Genomes (KEGG) pathway analysis using downregulated and upregulated DEGs upon B cells. **(E)** Box plots of the expression levels of GO biological process terms in B cells colored by donors of different conditions. **(F)** Significance checkup table for the percentage of each cell types and score of GO biological process terms in B cells. ns, non-significant. up, significantly higher or up-regulated. down, significantly lower or down-regulated.

As shown in **Figure 6A** and **Supplementary Figure 11**, the analysis of B cell functional subtypes showed that children appeared to have a lower proportion of naïve B cells and a higher proportion of activated B cells than adults in both healthy and diseased conditions (**Figure 6B**), suggesting an endogenous distinction of B cells between children and adults. DEGs analysis revealed that genes that may play a negative regulation on B cell response (e.g., *CDKN1A, NR4A2, JUNB*) were more abundantly expressed in children (**Figure 6C**). In accordance with the elevated proportion of activated B cells observed in children, the expression of the activation marker CD69 was found to be elevated in the P_MD group in comparison to the A_SC group (**Figure 6C**). Furthermore, we evaluated the expression of CD69 in B cell subsets of the P_MD and P_CR groups, with the latter group expectedly exhibiting a downregulated expression of CD69 in B cell subsets than the former group (**Supplementary Figure 12**). In addition, pathway analysis revealed that several KEGG pathways involved in B cell replication and differentiation (**Figure 6D**) and many GO biological processes (**Figures 6E, F**, **Supplementary Figure 13**) were downregulated. With all these findings, B cells in children with COVID-19 exhibit a markedly activated state, yet their response is conserved.

**Figure 7:**
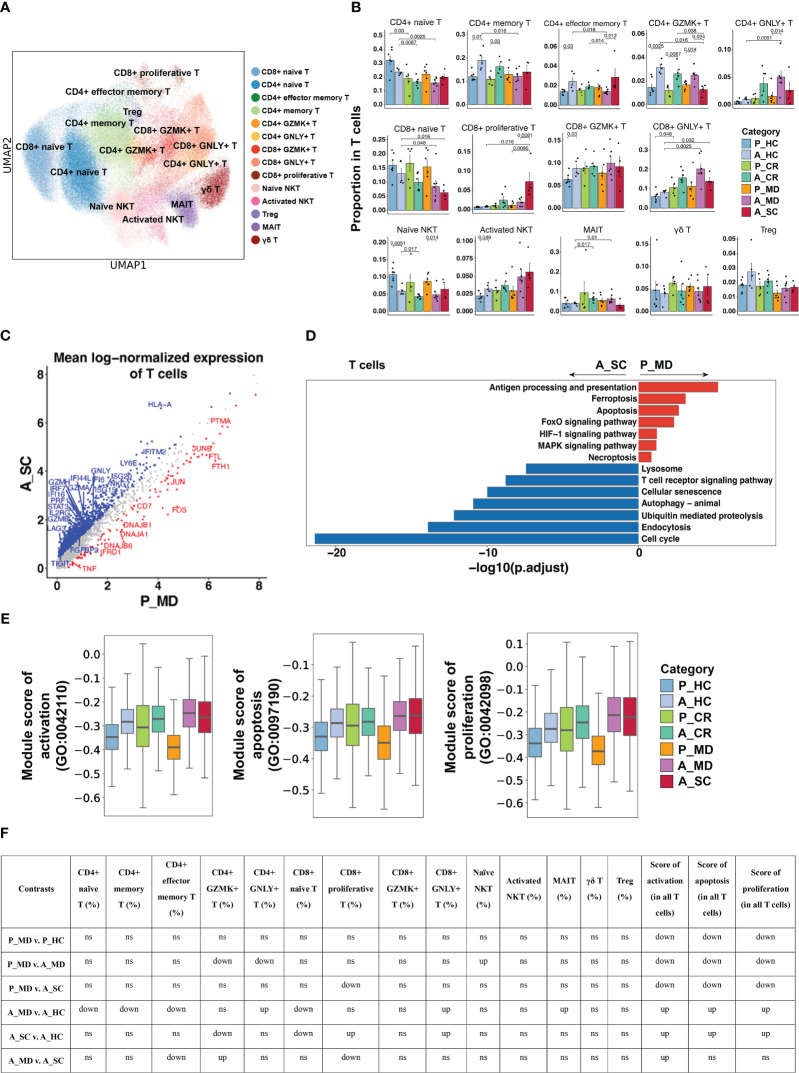
Characteristics of T cell phenotype in different conditions. **(A)** UMAP embedding of T cell colored by different subsets. **(B)** Bar plots showing the proportion of T cell subtypes colored by donors of different conditions. Error bars represent ± s.e.m. All differences with *P* < 0.05 are indicated; a two-sided unpaired Mann–Whitney *U*-test was used for analysis. P, pediatric; A, adult; MD, mild or moderate COVID-19; CR, convalescence; SC, severe or critical COVID-19; HC, healthy controls. **(C)** Scatter dot plot indicating DEGs in T cells between P_MD and A_SC group. The cutoffs for DEGs were set to log2(fold change) +- ≥ 0.5 with Benjamini-Hochberg adjusted *P* value < 0.01 (two-sided unpaired Mann–Whitney *U-*test). **(D)** Bar plot of Kyoto Encyclopedia of Genes and Genomes (KEGG) pathway analysis using downregulated and upregulated DEGs upon T cells. **(E)** Box plots of the expression levels of GO biological process terms in T cells colored by donors of different conditions. **(F)** Significance checkup table for the percentage of each cell type and score of GO biological process terms in T cells. ns, non-significant. up, significantly higher or up-regulated. down, significantly lower or down-regulated.

14 T cell subtypes (**Figure 7A**) were identified based upon functional cell marker gene expression (**Supplementary Figure 14**). It was observed that CD8+ naïve T cells decreased from P_MD group to A_MD group and A_SC group, possibly indicating a downward trend with age and disease severity (**Figure 7B**). The proportions of GZMK- and GNLY-expressing CD4+ T cell subtypes in P_MD group were significantly lower than that of A_MD group (both *p* = 0.014) (**Figure 7B**), suggesting a less-activated T cell response in children. In addition, downregulated cytotoxicity-associated genes (e.g., *GZMA, GZMB, GZMH* )(**Figure 7C** ), suppressed T cell receptor signaling pathway (**Figure 7D**), and lower gene set scores of activation, proliferation, and apoptosis GO terms (**Figures 7E, F**, **Supplementary Figure 15**), were all suggestive of a less active T cell response in pediatric COVID-19 (P_MD group). In contrast, both cytotoxicity- and exhaustion-associated genes were abundantly expressed in the severe/critical adult COVID-19 (A_SC) (**Supplementary Figure 16**), indicating an excessive and uncontrollable T-cell response in these patients. Hence, the T cell response in children with COVID-19 was not as strongly boosted as seen in adult counterparts.

## Discussion

The rapid spread of SARS-CoV-2 poses a constant threat to human health around the world, and the emerging new variants are still fueling a future pandemic. It’s reassuring to know that most children with COVID-19 require no or shorter hospitalizations and less respiratory support compared with adults (37). The dominating factor contributing to this phenomenon, though unknown yet, may be related to the immune response (37). As reported, adults with severe COVID-19 were considered to have a loss of “immune synchrony”, characterized by T cell lymphopenia, exhausted IFN-mediated signaling, and excessive myeloid cell activation (38, 39). In contrast, we observed restrained immune responses in children with the disease, as characterized by modest T/B cell response, conservative IFN-I signaling, suppressed monocyte activation, and robust NK cell cytotoxicity. Taken together, it might be assumable that the enhanced cytotoxic NK cells and less-activated monocytes in children with COVID-19 could confer competent early viral clearance while avoiding excessive inflammatory damages, thus resulting in a less pronounced activation of adaptive immunity later in the infection. In comparison, the incompetent and exhausted innate immunity in adult patients required greater assistance of subsequent adaptive immunity to accomplish viral clearance. Hence, the differences in innate immunity between children and adults may be a critical immunologic factor contributing to the distinct clinical presentations.

Monocytes could release a distinct set of chemokines and cytokines upon activation (40–42), and are closely associated with the progression of COVID-19 (39–41, 43). Since cytokine profiles observed in severe COVID-19 cases were similar to those observed in macrophage activation syndrome (MAS) (44), it might be assumable that the uncontrolled monocyte response is the dominating factor contributing to the COVID-19-associated hyperinflammation, which is further supported by the less activated monocytes of children in our findings. Importantly, CD14+ monocytes are crucial producers of IFN-I in various infections (34), which partially explains the suppressed ISG expression in children. In consistent with our findings, plenty of studies have proven that severe COVID-19 was characterized by enhanced IFN-I signaling (2, 45–49), which is seemingly contradictory to the well-known protective role of it. Actually, the uncontrolled IFN-I response has been proven to be associated with various infections, autoimmune and inflammatory diseases (50, 51). Concerning the underlying mechanism, Lee et al. found that IFN-I could induce hyperinflammation by recruiting monocytes and macrophages in mouse models of COVID-19 (52). Moreover, they revealed an “IFN-I-potentiated TNF/IL-1β inflammatory response” in severe/fatal COVID-19 using scRNA-seq analysis (45), which further clarified the pro-inflammatory role of IFN-I. Notably, the IFN-I response is also a phenotype of senescence and contributes to the maintenance of the senescence-associated secretory phenotype (SASP) (53). Cecco et al. recently found that using lamivudine could suppress age-associated inflammation (also called inflammaging) in aged mice by downregulating IFN-I response (53). Hence, a multi-dimensional regulation is critical to shape the overall outcome of IFN responses to achieve the optimal balance between protective immunity and inflammation-associated tissue damage. Collectively, there is a strong correlation (potentially causal) between an exaggerated IFN-I response and hyperinflammation, with aging possibly serving as a significant contributing factor. This concept helps to elucidate why the elderly population is more prone to cytokine storms and severe illnesses in light of the overall mildness of children with COVID-19.

As we know, NK cells are the main “killers” responsible for eliminating senescent, virus-infected, pre-malignant, and other abnormal cells in the body, being the initial defense mechanism against pathogens in the innate immune system. NK cells carry out their cytotoxicity function in various ways, such as initiating cytotoxic degranulation (e.g., perforin and granzyme), releasing inflammatory cytokines (e.g., IFN-γ, TNF-α), expressing members of the tumor necrosis factor superfamily (e.g. FASL, TRAIL) and mediating antibody-dependent cell-mediated cytotoxicity (ADCC) (35, 54). Human NK cells can be further divided into two principal NK cell subsets: CD16dimCD56bright NK cells and CD16+ NK cells (55). The CD16+ NK cell predominantly mediates the killing of target cells by degranulation, whereas the CD16dimCD56bright NK cell mainly exhibits immunoregulatory and cytokine-producing capacity (56). Kramer et al. reported that patients with moderate COVID-19 showed larger numbers of total NK cells and subsets than severe cases (57). However, in our study, we observed only an up-trend (not statistically significant) of the proportions of total NK cells and CD16dimCD56bright NK in P_MD group compared with A_MD and A_SC groups, with the discrepancy possibly due to different methods of calculation between their work and ours (absolute number vs. proportion). Nonetheless, we found remarkable functional differences between NK cells of pediatric and adult patients, including enriched TNF signaling pathway (mainly refers to CD16dimCD56bright NK cells; **Figure 5D**) and stronger degranulation (primarily refers to CD16+ NK cells; **Figures 5E, F**). In consistence, patients with severe COVID-19 were reported to be characterized by persistent depletion and reduced cytotoxic capacity of NK cells (49, 57, 58). Hence, the competent cytotoxic response of NK cells in children with mild/moderate COVID-19 may play a key role in the viral clearance during the early phase of the infection.

Notably, the cytotoxicity of NK cells seems to be impaired with age. For instance, Zheng et al. revealed an increase in expanded low-cytotoxic NK subsets and decreased virus defense responses in the older group (59), which explains the strong association between the decline in NK cell cytotoxicity and increased incidence of infectious diseases and cancers in older people (60). The identification of the key factors that promote NK cell senescence is of significant importance in regulating the killing function and thus improving the clinical prognosis of various diseases. The Notch gene, first identified in 1917, is highly conserved throughout evolution and plays a pivotal role in many physiological and pathological processes, including cell proliferation and migration, the immune response, angiogenesis, and so forth (61). Importantly, Notch signaling was also associated with cellular senescence, such as mediating the senescence of esophageal keratin-forming cells, which in turn regulates esophageal squamous carcinoma (62), driving the senescence of hepatic sinusoidal endothelial cells, which then inhibits hepatic repair after liver injury (61). Moreover, in 2019, Teo et al. identified Notch signaling as a key mediator of secondary senescence. They proved that the inhibition of Notch signaling may delay or even prevent the onset of secondary senescence in various cell types (36). In the case of NK cells, although Notch signaling has been found to be associated with NK cell development (63), its role in NK cell senescence remains not fully elucidated. Hence, the downregulated Notch signaling in NK cells of pediatric patients in our study, which coincided with the robust cytotoxicity and suppressed senescence of their NK cells, may suggest a novel NK cell senescence-associated factor and provide insights into the potential of NK cell-based immunotherapy on infectious diseases.

To be added, one of the dominating mechanisms for NK cells to distinguish healthy cells from diseased cells were the interactions between KIR and HLA-C (64). Specifically, HLA-C1 is the preferred ligand for the inhibitory receptors KIR2DL2 and KIR2DL3, while HLA-C2 is commonly recognized by KIR2DL1 (65). The inhibitory capacities of HLA-C-reactive KIR are associated with the progression of viral infection. For example, Lee et al. found that blocking KIR2DL1 enhanced the cytotoxicity of NK cells against SARS-CoV-2-infected cells in *in vitro* experiments (66). Khakoo et al. stated that the weaker inhibition conferred by KIR2DL3/HLA-C1 was found to be protective in acute Hepatitis C Virus infection, possibly by facilitating stronger NK cell responses (67). These findings seemingly indicate an unfavorable role of KIR2DL1 signaling by inhibiting NK cell cytotoxicity. However, several studies have reported that NK cells lacking inhibitory receptor-mediated signaling are hyporesponsive against target cells (56, 68). Moreover, both Bari et al. and Yawata et al. reported that stronger KIR2DL1 signaling exhibits significantly more degranulation and IFN-γ secretion than weaker KIR2DL1 signaling (69, 70). Also, Bari et al. further confirmed that stronger KIR2DL1 signaling was more potent than the weaker in licensing NK cells, resulting in better cancer control and patient survival after transplantation (70). Also, it is worth mentioning that the *in vivo* environment is intricate, and the functional state of NK cells is not a simple “on/off” switch but relies on the coordinated interplay of all NK cell activating/inhibiting receptors and corresponding ligands. In this way, it seems one-sided to regard KIR2DL1 signaling as only an inhibitory factor for NK cell cytotoxicity, as it might also be a prerequisite and guarantee of competent NK cell responses, which partly explains the remarkably higher expression level of KIR2DL1 gene in P_MD group (**Supplementary Figure 10**).

The maintenance of CD8+ naïve T cells is essential for effective antiviral defense, and a decrease in these cells is a key feature of immunosenescence in older people (71). Hou et al. found that aged patients with severe COVID-19 showed a decreased proportion of naïve CD8+ T cells and memory CD8+ T cells (72), which was consistent with our findings. Amrute et al. observed that T cell subsets displayed increased expression of effector activation markers in fatal COVID-19 patients (49). Hence, the abundant naïve CD8+ T cells and limited cytotoxic T cells in children could be more effective in restraining viral invasion while avoiding uncontrolled exhaustion and functional impairment.

Activation of B cells induces dynamic changes to get themselves into specific lymph tissue subcompartments for further activation and differentiation (73). Upon infection, B cell activation mainly leads to rapid induction of extrafollicular (EF) and slower development of germinal center (GC) responses in draining lymph nodes (73). EF responses enable B cells to differentiate into short-lived plasmablasts (SLPC) that primarily contribute to viral clearance, while GC response mainly aims to generate and maintain immunological memory by producing memory B cells and long-lived plasma cells (LLPC) (74). According to our findings, the activated B cells of children were kept at a higher level at both healthy and diseased states than their adult counterparts, but we didn’t observe remarkable increases in the proportions of plasma cells and memory B cells of children. As reported, activated B cells can also act as antigen processing cells (APCs) to carry antigens directly from sites of infection to lymph tissue (75, 76). Moreover, activated B cells are able to facilitate the interaction with activated T cells in the T:B border of lymph tissues, and assist the interactions of B cells with macrophages for antigen signals, leading to optimal humoral responses (75, 76). In summary, activated B cells exhibit indispensable regulatory roles in nearly all stages of immune responses, particularly in antigen presentation and lymphoid tissue remodeling. To be noted, in mouse models of lymphocytic choriomeningitis virus (LCMV) infection, IFN-I signaling has been proven to promote naïve B cells to differentiate into SLPCs, resulting in a lack of activated B cells (74). This is in line with our findings that mildly/moderately ill children showed inhibited IFN-I response, higher activated B cells, and lower plasma cells compared with severely/critically ill adults. Collectively, the endogenous higher activation of B cells in children, possibly resulting from the restricted IFN-I signaling, may be beneficial in orchestrating the overall immune response of children with COVID-19.

Concerning the antibody response, we have previously detected the dynamics of the amount and neutralization/titer of SARS-CoV-2-specific antibodies in serum samples from children with COVID-19 in other papers of our team (7, 15, 21), the specimens of which included those used in this study. We found that children with mild/moderate COVID-19 developed timely, protective, and organized SARS-CoV-2 S-RBD/N protein-specific IgM/IgA/IgG (7, 15, 21). Moreover, the seroconversion time of antibodies of children was earlier than that of adult patients according to the papers published during the same period (15, 77). Furthermore, Kim et al. recently confirmed that children exhibited higher SARS-CoV-2-specific IgG levels and better neutralization activity compared with adults following SARS-CoV-2 infection or vaccination (17). In comparison, severe COVID-19 patients showed strong but incompetent antibody responses, resulting in viral persistence and antibody-dependent enhancement (ADE) (39, 78, 79). Combining the past research and our results, it appears that COVID-19 patients exhibit varying patterns of humoral response based on age and disease severity. For instance, both mildly/moderately ill children and adults showed low proportions of plasma cells, but the former exhibited higher levels of protective antibodies than the latter, while severely/critically ill adults displayed a higher proportion of plasma cells but incompetent antibodies. These raise the question of what contributes to the different patterns of humoral response in various patients. It was reported that pediatric cases had a reduced breadth of antigen-specific antibodies compared to adults (40). Thus, besides of the amounts of plasma cells and antibodies, the targeted epitopes of antibodies might also affect the final protection of antibodies. Given that COVID-19 convalescent plasma treatment has been proven to be an effective treatment option and post-exposure prophylaxis (80), whether recombinant antibodies based on the pediatric antibody profile could lead to better therapeutic outcomes deserves further studies.

Along with aging, the immune system undergoes dramatic changes (11). However, the phenomenon of children having better clinical outcomes doesn’t happen in most other viral or bacterial infections, suggesting that aging-related immunological changes couldn’t fully explain why children are overwhelmingly spared from severe COVID-19. Hence, it is worthwhile to conduct further research on the factors leading to such a unique immune response in children with the disease, which might help facilitate the development of immunological treatments.

There are certain shortcomings in this study. First, our preliminary study had a relatively small sample size. Nevertheless, nonparametric statistical analyses were applied in the majority of key comparisons, and confirmatory validation assays such as the CD107a degranulation assay and *in vitro* IFN-stimulation assay were performed to ensure the accuracy of the primary conclusions drawn in the study. Second, some of latent batch effect in the child and adult data sets, which were generated in different facilities at different times (although these samples were sequenced and processed in the same way), might not be removable even by robust statistical batch removal procedures. Nonetheless, we have performed effective batch correction and integration between child and adult datasets to ensure the comparability of these scRNA-seq data. Third, our study was primarily in a case-control format that were not focused on mechanistic exploration of observed immune signatures in children with COVID-19, which is what we hope to pursue in future studies.

In conclusion, this preliminary study demonstrated distinct cell frequencies and activation status of major immune cell types of children with COVID-19. For the first time, this study at single cell resolution revealed specific immunological characteristics of children with the disease, including restrained IFN-I response, relatively less activated CD14+ monocytes, more activated NK cells with enhanced cytotoxicity, and suboptimal adaptive B and T cell activation and functions, which, particularly more robust NK cell cytotoxicity in PBMC, might help protect children from severe COVID-19.

## Data availability statement

The raw sequence data have been deposited in the Genome Sequence Archive in National Genomics Data, China National Center for Bioinformation/Beijing Institute of Genomics, Chinese Academy of Sciences accession number HRA006895 (https://ngdc.cncb.ac.cn/gsa-human/browse/HRA006895). The remaining data and source codes are available within the article, supplementary information, or available from the authors upon request.

## Ethics statement

The studies involving humans were approved by the Ethics Committee of Children’s Hospital of Fudan University [NO. (2020)27]. The studies were conducted in accordance with the local legislation and institutional requirements. Written informed consent for participation in this study was provided by the participants’ legal guardians/next of kin.

## Author contributions

RJ: Data curation, Formal analysis, Methodology, Project administration, Validation, Writing – original draft, Writing – review & editing. SH: Methodology, Software, Visualization, Writing – original draft. ZL: Methodology, Software, Visualization, Writing – original draft. HC: Data curation, Writing – review & editing. MZ: Data curation, Supervision, Writing – review & editing. PL: Data curation, Writing – review & editing. LL: Data curation, Writing – review & editing. MX: Data curation, Writing – review & editing. XZ: Conceptualization, Formal analysis, Funding acquisition, Investigation, Project administration, Resources, Supervision, Validation, Visualization, Writing – review & editing. MQ: Conceptualization, Funding acquisition, Investigation, Methodology, Project administration, Resources, Software, Supervision, Validation, Visualization, Writing – review & editing. JX: Conceptualization, Data curation, Formal analysis, Funding acquisition, Investigation, Methodology, Project administration, Resources, Supervision, Validation, Visualization, Writing – review & editing.
