# Supplementary material for: Immunological characterization and comparison of children with COVID-19 from their adult counterparts at single-cell resolution
Source: Front Immunol. 2024 Aug 1;15:1358725. doi: 10.3389/fimmu.2024.1358725 (PMC11325098; doi:10.3389/fimmu.2024.1358725)
Supplement: Supplementary file 6 [file DataSheet_6.docx]

**Supplementary Materials for**

**Immunological characterization and comparison of children with COVID-19 from their adult counterparts at single-cell resolution**

Ran Jia^1†^, Shiwen Hu^2†^, Zifeng Li^2†^, Hailing Chang^3^, Mei Zeng^3^, Pengcheng Liu^1^, Lijuan Lu^1^, Menghua Xu^1^, Xiaowen Zhai^2*^, Maoxiang Qian^4*^, Jin Xu^1, 5*^

**^*^Corresponding author:**

Jin Xu, jinxu_125@163.com

Maoxiang Qian, mxqian@fudan.edu.cn.

Xiaowen Zhai, xwzhai@fudan.edu.cn.

This file contains Supplementary Figure S1 to 16

**Guidelines for Diagnosis and Treatment of COVID-19:**

All the patients in this study were classified strictly following the Guidelines for Diagnosis and Treatment of COVID-19 issued by the National Health Commission of China (10th edition). Briefly, if the patients were mainly manifested as upper respiratory tract infections, they were categorized as mild illness. When the patients had persistent hyperpyrexia (> 3 days), cough/polypnea, radiographical findings of typical COVID-19 pneumonia, but normal respiratory rates (RR) (< 30/min), they were categorized as moderate illness. When adult patients had polypnea (RR ≥ 30/min), decreased PaO2/FiO2 (≤ 300 mmHg), progressively exacerbated clinical presentations, they were defined as severe illness, while for pediatric patients, persistent hyperpyrexia (> 3 days), polypnea, nasal flaring, triple concave sign, wheezing, etc., were indicative of severe illness. Patients with respiratory failure, shock, or organ dysfunction were defined as critical illness.

**Supplementary Figure S1**

**Supplementary Figure S1. UMAP embedding of PBMCs** **from all samples colored by donors of different conditions.** P, pediatric; A, adult; MD, mild or moderate COVID-19; CR, convalescence; SC, severe or critical COVID-19; HC, healthy controls.

**Supplementary Figure S2**

**Supplementary Figure S2. Hierarchical clustering of child and adult datasets.** Pseudo-bulk count matrices were generated using single-cell data, and unsupervised hierarchical clustering analysis were separately conducted within the adult and pediatric groups. P, pediatric; A, adult; HC, healthy controls; CR, convalescence; Mild, mild COVID-19; Moderate, moderate COVID-19; Severe, severe COVID-19; Critical, critical COVID-19.

**Supplementary Figure S3**

**Supplementary Figure S3. Violin plots presenting the distribution of classical cell markers for specific cell types.**

**Supplementary Figure S4**

**Supplementary Figure S4. Box plots of the expression levels of response to IFN in different cell types colored by donors of different conditions.** P, pediatric; A, adult; MD, mild or moderate COVID-19; CR, convalescence; SC, severe or critical COVID-19; HC, healthy controls.

**Supplementary Figure S5**

**
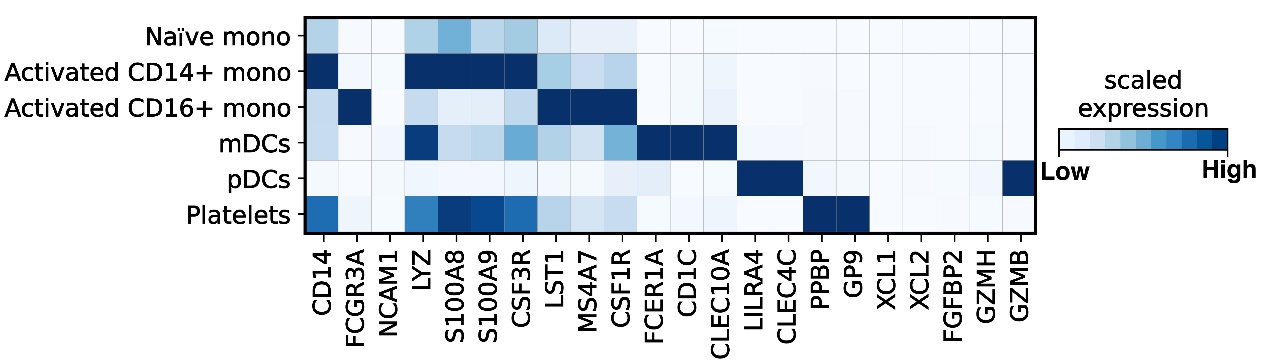
**

**Supplementary Figure S5**. **Heatmap of classical cell markers for each cell subtype in myeloid cells.**

**Supplementary Figure S6**

**Supplementary Figure S8**. **Heatmap of classical cell markers for each cell subtype in NK cells.**

**Supplementary Figure S7**

**Supplementary Figure S7**. **Box plots of the expression levels of GO biological process terms in myeloid cells colored by donors of different conditions.** P, pediatric; A, adult; MD, mild or moderate COVID-19; CR, convalescence; SC, severe or critical COVID-19; HC, healthy controls.

**Supplementary Figure S8**

 **Monocytes pDCs**

**Supplementary Figure S8. Heatmap of Toll-like receptor (TLRs) and interferon regulatory factors (IRFs) in monocytes (left) and plasmacytoid dendritic cells (pDCs) (right).**

**Supplementary Figure S9**

**Cytotoxic-associated genes Exhaustion-related genes**

**Supplementary Figure S9.** **Heatmap of cytotoxic-associated genes (left) and exhaustion-related genes (right) for NK cells in donors of different conditions.**

**Supplementary Figure S10**

**Senescence-associated genes Notch-signaling-associated genes**

**Supplementary Figure S10. Heatmap of senescence-related genes (left) and Notch-signaling-associated genes (right) for NK cells in donors of different conditions.** P, pediatric; A, adult; MD, mild or moderate COVID-19; CR, convalescence; SC, severe or critical COVID-19; HC, healthy controls.

**Supplementary Figure S11**

**Supplementary Figure S11.** **UMAP of classical cell markers expression in B cell subsets.**

**Supplementary Figure S12**

**Supplementary Figure S12.** **CD69 expression in B cell subsets of P_MD and P_CR groups.**

**Supplementary Figure S13**

**Supplementary Figure S13. Box plots of the expression levels of GO biological process terms in B cell subsets colored by donors of different conditions.** P, pediatric; A, adult; MD, mild or moderate COVID-19; CR, convalescence; SC, severe or critical COVID-19; HC, healthy controls.

**Supplementary Figure S14**


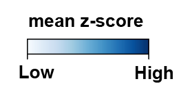


**Supplementary Figure S14.** **Heatmap of classical cell markers for T cell subsets.**

**Supplementary Figure S15**

**Supplementary Figure S15. Box plots of the expression levels of GO biological process terms in T cell subsets colored by donors of different conditions.** P, pediatric; A, adult; MD, mild or moderate COVID-19; CR, convalescence; SC, severe or critical COVID-19; HC, healthy controls.

**Supplementary Figure S16**

**Cytotoxic-associated genes Exhaustion-related genes**

**Supplementary Figure S16. Heatmap of cytotoxic-associated genes (left) and exhaustion-related genes (right) for T cells in different conditions.** P, pediatric; A, adult; MD, mild or moderate COVID-19; CR, convalescence; SC, severe or critical COVID-19; HC, healthy controls.
